# Inclusion of Refugee Peers – Differences Between Own Preferences and Expectations of the Peer Group

**DOI:** 10.3389/fpsyg.2022.855171

**Published:** 2022-04-08

**Authors:** Hanna Beißert, Kelly Lynn Mulvey

**Affiliations:** ^1^Department for Education and Human Development, DIPF | Leibniz Institute for Research and Information in Education, Frankfurt am Main, Germany; ^2^Department of Psychology, North Carolina State University, Raleigh, NC, United States

**Keywords:** refugees, social inclusion, inclusion decisions, adolescents, reasoning

## Abstract

Given the high numbers of refugees from Syria entering Germany in the recent years, the social integration of refugee youth has become an increasingly important issue in Germany. Thus, the current study examines adolescents’ decisions and reasoning around the inclusion of Syrian peers in Germany. Using a hypothetical scenario, we assessed adolescents’ (*N* = 100, *M* = 13.65 years, SD = 1.93, 51 females, 49 males) peer inclusion decisions and reasoning with attention to comparing inclusion of a Syrian refugee peer and a German peer. Given the importance of group norms for adolescents, we assessed not only adolescents’ own inclusion decisions, but also what they would expect their peer group to decide and what they think their peer group *should* do. Moreover, adolescents’ underlying reasoning was assessed. The analyses revealed that adolescents thought they would be more inclusive of a Syrian peer than a German peer and that their peer group should be more inclusive of a Syrian peer than a German peer. These tendencies toward including refugees were justified with references to morality as well as social-conventions. In contrast to their own decisions and to what they think their peer group *should*, participants expected their group would be more inclusive toward a German peer than a Syrian peer. This was mainly justified by referencing aspects of group functioning and psychological information about the peers, whereas moral and prosocial reasoning was very rarely used for the expected group decision. In sum, these findings document that adolescents in Germany wish to be inclusive regarding refugee peers and that they balance attention to morality and other domains of social reasoning when thinking about inclusion decisions while they expect that their peers will not consider morally relevant information when making these decisions. These findings have important practical implications as they indicate the importance of interventions that focus on promoting inclusive peer group norms.

## Introduction

In the recent years, refugee migration has increased tremendously all over Europe. Since the beginning of the Syrian civil war in the year 2011 almost 7 million people have fled to Europe (UNHCR, 2021), among them many children and adolescents (Eurostat, 2021). Moreover, estimates indicate that over one half of Syrian refugees in Germany are youth under age 18 (Eurostat, 2021). Consequently, in many European countries, the integration of refugees has become an increasingly important issue. However, integration is not a unidirectional process that can be accomplished by the refugees alone. Integration is a reciprocal process of mutual accommodation between the incoming refugees and the members of the host society (Berry et al., 2006). The members of the host society need to be open to integration and welcoming toward the refugees (Berry, 2011). Thus, the attitudes of the members of the host society toward refugees are crucial for integration. This does not only hold for formal aspects of integration such as educational or occupational opportunities, but also in terms of the social integration of refugees. The current study focuses on the openness of adolescents in Germany to include refugee peers from Syria into their peer activities. Additionally, the current study examines not only adolescents’ perceptions, but also their expectations for their peers’ inclusivity, given that adolescents may be influenced by their perceptions of their peers’ attitudes (Mulvey et al., 2014b). Finally, the current study examines not just evaluations, but also reasoning in order to explore underlying motivations that may drive inclusive practices toward refugees.

### The Need to Belong

Being included in peer activities is central for youth because the need for relatedness and social belonging are fundamental for human beings (Baumeister and Leary, 1995; Deci and Ryan, 2000) and fulfilling this need is considered essential for healthy development. For instance, feeling included or connected to others is associated with better health outcomes (Walton and Cohen, 2011), subjective well-being (King, 2015; Schmidt et al., 2020) and life-satisfaction (Rodríguez-Meirinhos et al., 2020). Additionally, belonging in class affects academic outcomes such as motivation (Walton et al., 2012), engagement (Furrer and Skinner, 2003; King, 2015), and achievement (Buhs and Ladd, 2001; Martin and Dowson, 2009) and additionally can buffer the negative effects of being bullied at school (Marksteiner et al., 2020). Being excluded in contrast, can have severe consequences for an individual’s health and well-being (Mulvey et al., 2017).

While a desire for connection with others is present even during infancy, as demonstrated by the research on the importance of secure attachments (Ainsworth, 1978), during adolescence, when peer relations become increasingly important, the need to belong and to be accepted by others is particularly strong (Jose et al., 2012; Lamblin et al., 2017). While adolescents in this phase strive for independence from parents, the peer group and reliable relationships with peers become increasingly important (Masten et al., 2009; Morningstar et al., 2019). Moreover, during adolescence, youth may feel pressure to conform to their peers’ expectations, behaviors, and attitudes in order to “fit in” (Brown et al., 1986; Miyajima and Naito, 2008; Choukas-Bradley et al., 2015; Mulvey and Killen, 2016b). Thus, examining adolescents’ tendencies toward inclusion, as well as how their own inclusivity might or might not align with their expectations of their peers’ inclusivity may provide particular insight into how to best support adolescents as they seek to belong and to build social connections with others. In fact, prior research demonstrates that even though adolescents do not always believe their peer group will be inclusive, they often place a high priority on preventing harm to others and assert that they, individually, will include others even if their group would not (Mulvey et al., 2014b; Mulvey and Killen, 2016b).

### The Special Situation of Refugee Youth

For youth from refugee families, social contacts are particularly important: refugee youth in Germany note challenges with friendships as a concern, but also highlight social support (for instance from friends and family) as central to coping with challenges they face (Alhaddad et al., 2021). Children and adolescents from refugee families experience high levels of trauma, and upheaval, with reports indicating that more than one third of asylum seeking youth in Germany meet the criteria for Post-Traumatic Stress Syndrome and 30% experience clinically significant bouts of depression (Müller et al., 2019). They had to leave their homes and their country and many of them have experienced traumatic events or other psychological stressors (Lustig et al., 2004; Ruf et al., 2010).

In such a precarious situation, stable social relationships are of particular importance (Alhaddad et al., 2021) and supporting a feeling of relatedness in early resettlement is essential for young refugees’ well-being (Correa-Velez et al., 2010). Thus, relatedness might be particularly important for adolescents from refugee families; and it is conceivable that social exclusion might have an even greater impact on them than on other groups. In line with this, research indicates that social exclusion during the acculturation process is a significant acculturative stressor, making integration more difficult (Verkuyten and Thijs, 2002; Ward et al., 2020) and that being included (i.e., having friends at school) can serve as a key coping mechanism during the acculturation process (Alhaddad et al., 2021).

However, research has shown, that many refugees face social exclusion and marginalization when coming to a new country (Beirens et al., 2007; Kocak et al., 2021). This tendency to exclude refugees may be rooted in children’s essentialist thinking about national identity (Feeney et al., 2020). Children begin to think of national identity as immutable quite early and this essentialist thinking about nationality is quite strong, having been documented in many different countries (Hussak and Cimpian, 2019; Davoodi et al., 2020; Feeney et al., 2020; Siddiqui et al., 2020). Thus, to improve the situation of refugee youth, one aim should be to support them and provide opportunities to build friendships with local peers in order to foster positive connections and develop relationships in their host society, especially at school (Marshall et al., 2016), especially given that they may not be seen as part of the host society. As mentioned above, integration is a reciprocal process and the openness of the members of the host society is very important for this (Berry et al., 2006; Berry, 2011). Thus, one key step in ensuring that refugee youth have ample opportunities to build relationships and connections in the new country is to focus on understanding the attitudes and reasoning of youth from the host society around inclusion of refugee peers.

Examining this question in Germany, in particular, is important given the high number of refugee youth in Germany (Eurostat, 2021). Further, prior research on German adolescents’ attitudes toward refugees documents that more German adolescents perceived that they learned about the cultural history and traditions of both Germans and refugees and the similar German adolescents saw themselves and refugees, the more prosocial they intended to be toward refugees (Aral et al., 2021). Additionally, prior research demonstrates that German adolescents were more likely to include Syrian refugees who had good German language skills, suggesting the importance of cultural integration for inclusivity (Beißert et al., 2020). While some prior research demonstrates that German youth may wish to be inclusive of refugees, and act in prosocial ways, much less is known about the underlying reasons youth use when making inclusion decisions.

### Theoretical Framework: Social Reasoning Development Perspective

Given our interest in understanding youth attitudes and reasoning, we framed this study using the Social Reasoning Development perspective (SRD; Rutland et al., 2010; Rutland and Killen, 2015). This perspective, which draws on social domain theory (Turiel, 1983; Smetana et al., 2014) and social identity theory (Tajfel and Turner, 1976, 1986), posits that individuals’ social decisions are often made as they balance information about group loyalty and group priorities with information about what is morally right and just (Rutland et al., 2010; Rutland and Killen, 2015). In fact, even very young children and infants demonstrate support for their ingroup (Jin and Baillargeon, 2017; Pun et al., 2018). Prior research has shown that many adolescents in Germany have an open attitude regarding refugees in Germany (Albert et al., 2019) with more open attitudes the younger they are (Kober and Kösemen, 2019). Additionally, children and adolescents in Germany are generally quite open to include refugees in their peer activities (Beißert et al., 2020; Andresen et al., 2021). However, prior research from a SRD perspective documents that when youth must make decisions between inclusion of an in-group or an out-group member (for instance, a German peer or a Syrian peer), at times they do prioritize inclusion of in-group members and justify these choices by referencing group functioning and group loyalty (Mulvey et al., 2014a). Research also demonstrates, however, that moral principles do play a role in adolescents’ inclusion decisions, with findings suggesting that children and adolescents will reason about fairness, and harm when making inclusion decisions (Killen et al., 2013). As noted, at times there is also a disconnect between one’s own expectations of inclusion and their expectations of their group’s inclusivity (Mulvey et al., 2018). Findings also suggest that peers do often expect their ingroup to be less inclusive, and factors such as stereotypes can shape these expectations (Hitti and Killen, 2015b). Recent scholarship on intergroup attitudes toward refugees documents that children and adolescents struggle to take the perspective of refugees and immigrants and highlights how factors such as peer expectations can shape intergroup relations between native and refugee youth (Gönültaş and Mulvey, 2019). In fact, research suggests that even toddlers differentiate depending on context when evaluating situations involving helping others who are dissimilar to one’s self (Geraci and Franchin, 2021). Thus, the aim of the current study was to more comprehensively understand adolescents’ inclusive tendencies in a salient context: Germany, which hosts over a million refugees as of 2021 (UNHCR, 2021).

### Current Study

What is still unknown, however, is how adolescents make inclusion decisions for refugee peers and what underlying reasons they will use when making inclusion decisions. Further, much prior research on inclusion has used a forced choice paradigm where you must select between two peers (Hitti et al., 2014; Mulvey et al., 2014b; Hitti and Killen, 2015a). In the current study, participants were asked to indicate likelihood of inclusion for both a native and refugee peer and to provide reasoning for these evaluations in order to have a more complete picture of their reasoning and decisions.

Moreover, as demonstrated by prior research (Mulvey et al., 2014b,^2018^), it is not only important to ask adolescents what they personally would decide. Decisions and behavior are not only based on one’s personal norms, attitudes, or values, but group norms are very important as well and can influence adolescents’ decisions and behavior (Killen et al., 2017; Mulvey and Killen, 2017; McGuire et al., 2018). Children and adolescents may struggle with social decisions when group norms conflict with individual norms or values (Mulvey et al., 2013). Thus, we are not only interested in what adolescents, themselves, would decide. We are also interested in what they think what their peer group would decide, given the very powerful influence that the norms and decisions of the peer group can have on one’s behaviors and intentions. Therefore, we examined adolescents’ own decisions and compared them with what they expect their group to do and what they think their group should do. With these measures, we can assess individual decisions, expected group decisions and prescriptions about what adolescents believe is the right thing to do.

Our aim was to also explore the reasoning or justifications that underlie these decisions. The social domain model identifies three domains of social reasoning, the moral domain (justice and welfare), the social-conventional domain (conventions, traditions, and group norms), and the psychological domain (personal choice, psychological knowledge, and autonomy) (Turiel, 1983; Smetana et al., 2014). In the current research, all three domains play important roles: adolescents may consider the moral domain (e.g., feeling empathy or showing prosocial behavior), the social-conventional domain (e.g., aspects of group functioning or perceiving the pressure to show loyalty to the group and maintain the group norms) and the psychological domain (e.g., applying psychological knowledge or referring to personal choices or autonomy).

Thus, using a hypothetical scenario, we asked German adolescents to make judgments about their own inclusion of German and Syrian peers, as well as their expectations of their group’s inclusion and their sense of who should be included. We also asked them to provide reasoning for each assessment. We expected that:

1) Adolescents would expect that their group would be less inclusive of the Syrian peer than they would and then they thought their group should.
2) Adolescents who were more inclusive of the Syrian peer than the German peer would use more references to the moral domain, recognizing the importance of inclusion and prevention of harm of the Syrian refugees.
3) Adolescents would reason about the group decision using more references to social-conventions and group functioning and would reason about their own decision and their prescriptive decision for their group using more moral reasoning.
4) Adolescents would use less reasoning about the psychological domain (for instance autonomy) for the prescriptive group decision than their own decision or their expected group decision.

## Materials and Methods

### Participants

The study included 100 adolescents (*M* = 13.65 years, SD = 1.93) attending grades 5–10 of a high school (Gymnasium) in Northern Germany. The sample was approximately evenly divided by gender (51 female, 49 male) and 39% of the participants had a migration history in the family (i.e., at least one parent born in a country other than Germany). Three participants were excluded from the analyses as their families were from Syria, and thus, the in-group-out-group manipulation would not have worked for them as we used Syrian refugees as the focal out-group.

### Design and Procedures

Participants completed paper-pencil questionnaires in class under the guidance of a trained research assistant. Participation was voluntary and informed consent was obtained from all participants and their parents. Additionally, before handing out the questionnaires, the research assistant reminded participants about the voluntariness and anonymity of the participation and that there were no disadvantages if they decided not to participate or leave the study early without completing it. After the participants had completed the surveys, they were debriefed about the background of the study. They had the possibility to ask questions and talk with the research assistant about the aims and the background study.

### Materials and Measures

The survey included demographic questions (age, grade, migration history in the family) and a hypothetical scenario, in which the participants had to decide which of two peers they would like to include in a leisure time activity. They were told that they can invite only one more person. But there are two additional peers who would like to join the group. Both are new in class; one moved here from another German town and the other one came here with his family as refugee from Syria.

The exact wording of the vignette was as follows:

*Imagine you have a group of friends at school. You usually spend recess and much of your free time together. The following situation refers to this group.*

*Imagine you and your group are planning to play video games, this afternoon. You can only invite one other person. There are two boys/girls, who would like to join your group: Lukas/Laura and Rami/Shata. Both are new at your school. Lukas/Laura moved here from Frankfurt, he/she is German. Rami/Shata came to Germany with his/her family as a refugee from Syria.*

To avoid intergroup effects based on gender, the names of the protagonists in the scenario matched the gender of the participant.

After reading the scenario, the participants had to answer the following three questions for each protagonist separately: (1) How likely is it that you would choose xxx? (own decision) (2) What do you think, how likely is it that your group would choose xxx? (expected group decision) (3) Do you think, your group *should* choose xxx? (prescriptive group decision). Each of these three measures was presented on a separate page including the questions regarding both protagonists. The order of questions was the same for all participants. First, they responded to the question about the German peer, followed by the question about the Syrian peer. Participants answered all questions with a six-point Likert-type scale. For the questions (1) and (2), this scale ranged from 1 = very unlikely to 6 = very likely. For question (3), the scale ranged from 1 = not at all to 6 = definitely. For each measure, participants were also asked to provide reasoning about their choice (why?).

As participants assessed both inclusion of the German and Syrian peer, this manipulation was within subjects.

### Coding of Reasoning

To code participants’ answers to the open-ended questions (i.e., the reasoning about their decisions), a coding system was established drawing on prior research (Beißert et al., 2020) that was extended by adding categories inductively developed from the surveys themselves (see Table 1 overview and examples).

**TABLE 1:** Coding system and frequencies of usage.

	Own decision		Expected group decision		Prescriptive group decision		Total
	German	Syrian	German	Syrian	German	Syrian	
**MORAL DOMAIN**							
**Moral**							
*“because there should be fairness”*	15	17	2	4	18	25	81
**Prosocial**							
*“because I want to help her find friends”*	14	26	3	6	6	19	74
**SOCIAL-CONVENTIONAL DOMAIN**							
**Group functioning**							
*“it’s easier to play with someone who knows our culture”*	25	18	31	25	12	13	124
**Origin**							
*“I’d choose him because he is German”*	3	4	6	7	8	6	34
**PERSONAL DOMAIN**							
**Autonomy**							
*“because I want to get to know her”*	7	11	2	3	5	3	31
**Psychological information about skills/characteristics**							
*“if she is nice and friendly why should I not choose her”*	30	27	14	12	10	8	101
**Xenophobia and stereotypes**							
*“Black people don’t belong here”*	4	5	11	10	1	6	37
**Other**							
Useful, but single statements	17	10	7	12	7	3	56

Coders coded up to three relevant justifications for each statement. If the participant used only one code, this was assigned a value of 1.0. If they used two codes, each was given a value of 0.5. If three codes were used, each was given a value of 0.33. Coding was completed by two independent coders. Based on 25% of the interviews, interrater reliability was high, with Cohen’s kappa = 0.83.

## Results

Data were analyzed using repeated measures ANOVAs. As preliminary analyses revealed that there were no effects based on the participants’ own migration history (for inclusion decisions) and migration history, age and gender (for reasoning), these variables were not included in the respective analyses. Age was included as a covariate for the inclusion decisions just to confirm effects above and beyond age.

### Inclusion Decisions

**FIGURE 1:**
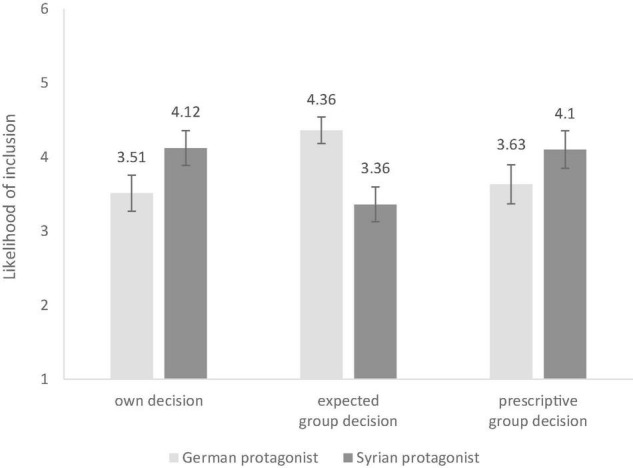
Inclusions decisions for both protagonists and all three measures. High values indicate a high likelihood include the respective protagonist.

To test for differences in inclusion decisions for the two protagonists and across the three questions, a 2 (gender: male, female) × 2 (protagonist: German, Syrian) × 3 (measure: own inclusion decision, expected group decision, prescriptive group decision) ANOVA was conducted with repeated measures on the last two factors with age as a covariate. There was a significant main effect of participant gender, *F*(1,89) = 5.920, *p* = 0.017, $\eta_{p}^{2}=0.06$, revealing that girls were slightly more inclusive than boys. Further, results revealed a significant interaction of protagonist and measure, *F*(1.42,125.96) = 12.70, *p* < 0.001, $\eta_{p}^{2}=0.12$. The Greenhouse–Geisser adjustment was used to correct violations of sphericity. Pairwise comparisons revealed that for the own decision and the prescriptive group decision, participants were more inclusive of the Syrian protagonist than the German protagonist. For the expected group decision in contrast, participants expected their group would be more inclusive to the German protagonist than the Syrian one. See Figure 1 for these results and Table 2 for the respective pairwise comparisons.

**TABLE 2:** Means and Standard Deviations of all three measures for each protagonist.

Measure	*M*_German_ (SD)			*M*_Syrian_ (SD)		
	Female	Male	Total	Female	Male	Total
Own decision	3.54 (1.64)	3.48 (1.72)	3.51^a,e^ (1.19)	4.42 (1.59)	3.81 (1.17)	4.12^c,e^ (1.15)
Expected group decision	4.38 (1.22)	4.34 (1.27)	4.36^a,b,f^ (0.88)	3.48 (1.63)	3.25 (1.70)	3.36^c,d,f^ (1.15)
Prescriptive group decision	3.65 (1.79)	3.61 (1.87)	3.63^b,g^ (1.29)	4.40 (1.72)	3.79 (1.80)	4.10^d,g^ (1.24)

*^a,b,c,d,f^p < 0.001, ^e^p = 0.003, ^g^p = 0.016.*

### Reasoning Analyses

Reasoning analyses were conducted on the proportional use of the four most used reasoning codes. These categories were “moral,” “prosocial,” “group functioning,” and “psychological information.” In order to test for differences in reasoning between the two protagonists and the three measures, a 2 (protagonist: German, Syrian) × 3 (measure: own inclusion decision, expected group decision, prescriptive group decision) × 4 (category: moral, prosocial, group functioning, and psychological information) ANOVA was run for proportional use of each code.

The analysis revealed a significant interaction between category and protagonist, *F*(2.45,178.59) = 6.47, *p* < 0.001, $\eta_{p}^{2}=0.08$, and a significant interaction of category and measure, *F*(5.17,377.52) = 8.26, *p* < 0.001, $\eta_{p}^{2}=0.10$. The Huynh–Feldt adjustment was used to correct violations of sphericity. The respective comparisons will be presented in the following two sections.

#### Differences in Category Use Based on the Two Different Protagonists

**FIGURE 2:**
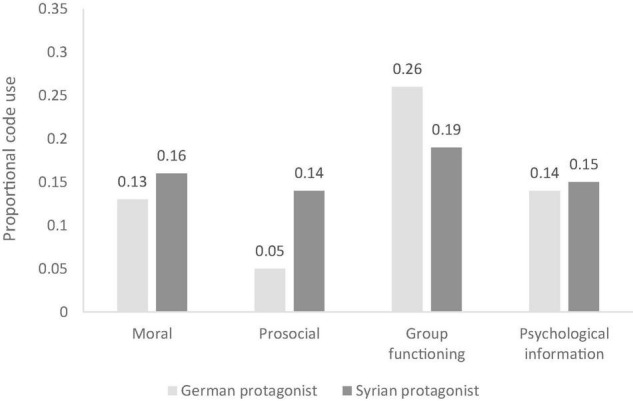
Category use for both protagonists across all three measures.

Pairwise comparisons revealed that justifications from the category “prosocial” were used more often when justifying the inclusion decision of the Syrian protagonist than when justifying the inclusion decision regarding the German protagonist, *p* < 0.001. Further, reasons related to group functioning were referenced more frequently when reasoning about the inclusion of the German protagonist compared to the Syrian protagonist, *p* = 0.007. See Figure 2 for means.

#### Differences in Category Use Based on the Three Different Measures

**FIGURE 3:**
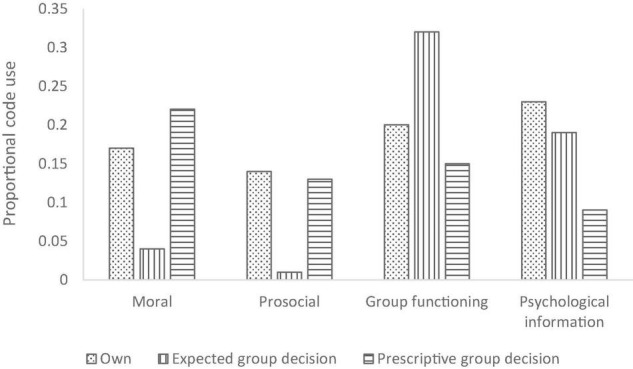
Category use for all three measures across both protagonists.

In terms of the interaction between measure and category, pairwise comparisons revealed that the categories “moral” and “prosocial” were used more often, when justifying the own decision and the prescriptive group decision than when reasoning about the expected group decision, *p*s ≤ 0.001. In contrast, justifications related to group functioning were used much more frequently in reasoning about the expected group decision than in reasoning about the own decision or the prescriptive group decision, *p*s < 0.05. Further, participants used the category “psychological information” more often when justifying their own decision compared to the expected group decision and or the prescriptive group decision, *p*s < 0.01. See Figure 3 for means.

## Discussion

The current study was conducted in Germany and examined adolescents’ peer inclusion decisions and reasoning with attention to comparing inclusion of a Syrian refugee peer and a German peer. Moreover, we assessed not only adolescents’ own inclusion decisions, but also what they would expect their peer group to decide and what they think their peer group *should* do. Additionally, we were interested in adolescents’ underlying reasoning. Our novel findings document that adolescents thought they would be more inclusive of a Syrian peer than a German peer and that one should be more inclusive of a Syrian peer than a German peer. These tendencies toward including refugees were justified with references to morality as well as social-conventions. On the other hand, participants expected their group would be more inclusive toward a German peer than a Syrian peer and justified these decisions primarily by referencing group functioning and psychological information about the peers. These findings document the important ways in which adolescents recognize the value of including refugees, but also acknowledge that the norms of their peer group may not support such inclusion.

On a positive note, we found that adolescents’ own decisions largely correspond with their prescriptive group decision, i.e., what they thought the group *should* do. However, while they thought one should and that they would include a Syrian refugee peer, they also believed that their group would be less likely to include a Syrian peer. Interestingly, the means for all responses, including the group decision, were near or above the mid-point, suggesting that participants generally had high expectations for their own inclusivity and their peers’ inclusive, even though they were significantly less likely to expect their group to include the Syrian peer. This is an important extension of prior research, which has often employed a forced choice inclusion paradigm (Hitti et al., 2014; Hitti and Killen, 2015a; Mulvey et al., 2018), and these findings indicate that, if possible, adolescents generally would like to include peers regardless of their background. This is important, given the findings that suggest how central inclusion is for adolescents’ well-being (Schmidt et al., 2020). However, although prior research does document that youth’s inclusion intentions do often align with their behaviors (Mulvey et al., 2018), findings also reveal that experiences of social exclusion are quite common (Killen and Rutland, 2011). For example, more than 25% of youth in the United States report experiencing repeated social exclusion (Wang et al., 2010). Thus, it may be that there is still a disconnect between adolescents’ desires to be inclusive and their actual behaviors. Our findings, then, are consistent with prior developmental theories, in particular the SRD perspective, that highlights the tension children and adolescents may feel between their moral principles that encourage inclusion and a desire to maintain connection to their group (Rutland et al., 2010; Rutland and Killen, 2015). Research on refugee youth in Germany notes that difficulties with friendships and social connections are a key challenge they face (Alhaddad et al., 2021), highlighting the importance of continued attention to fostering inclusive tendencies.

It may be that expectations of peer norms that promote including ingroup members over outgroup members may explain why social exclusion is still so prevalent. In the current study we do find that adolescents rate their peers’ inclusion of Syrian peers to be significantly lower than their own inclusion desires. Moreover, perceptions of exclusive peer group norms can be very powerful, even leading to greater exclusion when school norms promote inclusion (McGuire et al., 2015). Our findings align with prior research which demonstrates that expectations for one’s group and one’s own expected inclusion are often mis-aligned (Mulvey et al., 2014b,^2018^; Mulvey and Killen, 2016a). Taken together, these findings indicate that interventions that encourage the general inclusive tendencies of adolescents and promote norms of inclusivity may be effective. It is also important to note that our findings document a gender difference, with female participants generally reporting more inclusive tendencies than male participants, consistent with prior research (Killen, 2007; Beißert et al., 2020). Thus, interventions might also work to ensure that both boys and girls receive encouragement for inclusive behavior.

In terms of reasoning about inclusion decisions, our findings document nuances in adolescents’ reasoning, consistent with prior findings (Mulvey, 2015). Specifically, when reasoning about choosing to include a Syrian peer, adolescents used more moral and prosocial reasons, highlighting their recognition of the importance of fair treatment and helping refugee peers to connect with others. However, even when evaluating inclusion of a Syrian peer, adolescents referencing psychological information about that peer and even group functioning. Thus, they really did think about inclusion decisions of refugees in multifaceted ways. For example, an 11-year-old girl said “We should choose Shata because she might be nice and I want to get to know her. We can help her to get along in this new country. However, on the other side, we might have less fun playing with her because we need to explain and translate things all the time.” Interestingly, prosocial reasoning really only emerged when considering including the Syrian peer and not the German peer, suggesting that adolescents may recognize the challenges that Syrian refugees are facing (Marshall et al., 2016; Gönültaş and Mulvey, 2019; Alhaddad et al., 2021). This becomes apparent in statements like “Because he is a refugee and has not had such an easy life so far” (12-year-old boy) or “She fled from another country and now is sad because she probably had to leave many friends there” (11-year-old girl).

While adolescents reasoned about their own decision and what they should one should do, they used a range of different reasons, noting moral, prosocial, group functioning and psychological concerns. However, when considering how inclusive their peer group might be, adolescents tended to rely more on social-conventional and psychological reasoning. They asserted that their group might be concerned with how the group would operate if a Syrian peer was included, for instance. In fact, moral and prosocial reasoning was very rarely used for the expected group decision. This suggests that adolescents’ own decision-making balances attention to morality and other domains of social reasoning, while they expect their peers will not consider morally relevant information when making decisions. This suggests that interventions might focus on reasoning and giving adolescents opportunities to talk together about why it might be valuable to include others, with attention to issues around equity, fairness, and harm.

In concert, these findings suggest that adolescents do wish to be inclusive, and consider inclusion from a variety of standpoints. However, they also expect that their peers will be less inclusive than they individually would or than they should. These findings have implications for programs to promote inclusion, generally, as well as inclusion of refugee peers, in particular. Specifically, the results highlight the importance of encouraging adolescents to talk with each other about their desires to be inclusive, promoting norms of inclusion and helping each other to see the many benefits of being inclusive.

### Strengths and Limitations

The current study does provide important and novel findings. Namely, this study’s strengths include the rich assessment of adolescents’ reasoning and careful approach to asking participants to evaluate both their own and their group’s expected behaviors. Importantly, we document German adolescents’ inclusivity tendencies: they were generally quite inclusive and thought they would be more inclusive of a Syrian peer than a German peer and that one should be more inclusive of a Syrian peer than a German peer, highlighting their attunement to the challenges faced by refugee peers. While we extended prior literature by asking participants to provide separate evaluations and reasoning for each potential peer whom they might include, participants did, at times, mention both protagonists in their reasoning. This may suggest that they were still focused on the fact that there was only space for one peer and made their evaluations considering the relative likelihood of including one peer over another. This consideration of both peers may have participants to provide reasoning considering both inclusion of one and exclusion of the other. Without this blending of their evaluations, it is possible that the differences in reasoning would be more pronounced.

As noted, participants generally reported high rates of inclusion. This indicates that there may be a social-desirability effect at play. However, prior research showed that participants’ responses in hypothetical scenarios correspond with their authentic decisions in behavioral experiments (Mulvey et al., 2018), which provides support for the use of hypothetical scenarios in this context. This research explored adolescents’ reasoning, but we were not able to deeply examine developmental changes in adolescents’ evaluations. However, prior research in China documents that adolescents are often more exclusive than are young adults when considering inclusion of language out-group members (Zheng et al., 2021). We were also unable to examine the impact of intergroup contact with refugees, although prior research does demonstrate the importance of positive intergroup contact (Gönültaş and Mulvey, 2019). Thus, future research should aim to explore age-related patterns and the role of intergroup contact in shaping inclusivity toward refugees. Additionally, this research focused on participants from one school in Germany. Future research should aim to test the generalizability of these findings in different settings and contexts. Finally, this study only assessed inclusion in a leisure-time activity. However, refugees may also struggle with inclusion in other settings, for instance, in academic contexts. Future research should continue to explore inclusive tendencies in a range of contexts and settings.

## Conclusion

The current study documents adolescents’ decisions and reasoning around inclusion of German and Syrian peers, revealing the important ways in which adolescents’ own expectations differ from their expectations of their peer group’s inclusivity. Moreover, the findings reveal complexity in adolescents’ social reasoning. Adolescents generally expected their peers would focus more on group functioning when making inclusion decisions, but they recognized the importance of morality, prosociality, group functioning and even considered the psychological traits of the peers who they might include. In sum, the findings provide evidence that highlights the importance of interventions which work to promote inclusive peer group norms.

## Data Availability Statement

The datasets presented in this article are not readily available because the confirmed consent of the parents of the participants did not include that we share the data with other researchers. Requests to access the datasets should be directed to HB, beissert@dipf.de.

## Ethics Statement

Ethical review and approval was not required for the study on human participants in accordance with the local legislation and institutional requirements. Written informed consent to participate in this study was provided by the participants’ legal guardian/next of kin.

## Author Contributions

Both authors contributed substantially to the conception and design of the study and the manuscript. Both authors were involved in the planning of the analyses and interpretation of data. Both authors approve the final version to be published and agree to be accountable for all aspects of the work in ensuring that questions related to the accuracy or integrity of any part of the work are appropriately investigated and resolved.

## Conflict of Interest

The authors declare that the research was conducted in the absence of any commercial or financial relationships that could be construed as a potential conflict of interest.

## Publisher’s Note

All claims expressed in this article are solely those of the authors and do not necessarily represent those of their affiliated organizations, or those of the publisher, the editors and the reviewers. Any product that may be evaluated in this article, or claim that may be made by its manufacturer, is not guaranteed or endorsed by the publisher.
